# Determination and Analysis of Composition, Structure, and Properties of Teff Protein Fractions

**DOI:** 10.3390/foods12213965

**Published:** 2023-10-30

**Authors:** Zhenyang Quan, Lili Zhang, Wenping Chang, Xiangli Ding, Jianya Qian, Jianhua Tang

**Affiliations:** 1 School of Tourism and Culinary Science, Yangzhou University, Huayang Xilu 196, Yangzhou 225127, China; mx120211226@yzu.edu.cn (Z.Q.); mx120231282@stu.yzu.edu.cn (W.C.); dingxl@yzu.edu.cn (X.D.); 2 School of Food Science and Engineering, Yangzhou University, Huayang Xilu 196, Yangzhou 225127, China; mz120201830@yzu.edu.cn (L.Z.); jyqian@yzu.edu.cn (J.Q.); 3 Key Laboratory of Chinese Cuisine Intangible Cultural Heritage Technology Inheritance, Ministry of Culture and Tourism, Huayang Xilu 196, Yangzhou 225127, China

**Keywords:** teff, protein fractions, composition, structure, properties

## Abstract

To develop teff-based food products with acceptable quality, the composition, structure, and properties of teff protein fractions should be better understood. In this study, teff proteins were extracted, and their protein composition, structure, and properties were calculated, analyzed, and compared with those of wheat gliadin and glutenin. Results showed that teff flour contained 9.07% protein, with prolamin as its main protein fraction. The isoelectric points of albumin, globulin, prolamin, and glutelin were at pH 3.6, 3.0, 4.4, and 3.4, respectively. Teff prolamin and glutelin showed a significant difference in amino acids and free energy of hydration compared to wheat gliadins and glutenins. The protein chain length of teff prolamins was smaller than that of wheat gliadins, and teff glutelins lacked high molecular weight glutelin subunits. Teff prolamin had the highest α-helices content (27.08%), whereas no random coils were determined, which is different from wheat gliadin. Teff glutelin had a lower content of β-turn than wheat glutenin, and no α-helices were determined in it. Teff prolamin and glutelin had lower disulfide bond content and surface hydrophobicity. Teff prolamin had significantly higher thermal stability than wheat gliadin, whereas the thermal stability of teff glutelin was significantly lower than that of wheat glutenin.

## 1. Introduction

Teff is an indigenous Ethiopian annual crop [1]. Its flour is a typical nutritionally well-packed whole grain flour because the small grain size makes it only consumed with germ and bran included. It is rich in carbohydrates and minerals [2], with no gluten or gluten-like protein. These properties make it suitable for gluten-free food production. These characteristics, accompanied by the low GI of teff flour, result in growing global interest in developing teff-based food products. Teff has been successfully introduced and cultivated in many parts of the world, including the USA, Canada, Australia, Switzerland, and the Netherlands. Except for traditional Injera, porridge (muk), sweet, dry unleavened bread (kita), and local alcoholic drinks (tela and katikalla), non-traditional products, including cookies, cakes, pasta, bread, and fat replacer made with teff, were reported in recent years.

Teff is usually used in unleavened foods, such as cookies, pasta, and unleavened breads. For leavened bread, teff flour can only be used at a ratio of no more than 40% substitution in composite flour with wheat. Encompassing detrimental effects on bread’s physical and sensory quality would arise at high teff flour levels. Sourdough, microbial transglutaminase, hydroxypropyl methyl cellulose, and/or xanthans, and combinations of enzymes, must be used to maintain bread quality. There is no report on teff alkaline and white-salted noodles. That is because teff is one of the non-gluten-forming cereals, even though it has a comparable crude protein content to wheat flour. When used in traditional wheat-based foods, especially leavened bread and noodles, where the gluten matrix plays an essential role in structure formation and final product quality, it is a technological challenge to use teff at an extremely high level. Apart from the dilution effect on gluten of teff dietary fibers, which impairs proper dough development capacity during kneading in the dough system, the composition and characteristics of starches and proteins, the two most abundant components in teff, are crucial factors in teff flour processing difficulties. 

Currently, work has been conducted on the nutritional composition and characteristic property determination of teff flour and teff-based food product development. In contrast, studies on the fractionation and characterization of teff proteins are limited. Gliadin and glutenin are responsible for the unique viscoelastic properties of the dough [3]. It is proposed that the variations in gluten quality, dough properties, and quality of flour-based products depend on the balance between gliadin and glutelin [4]. Zhang et al. [5] analyzed the impact of globulin on dough properties and product qualities and found that the effect of globulin cannot be ignored. Wheat gliadin and glutenin make up 70% to 80% of wheat protein, contributing to the unique functional properties of wheat gluten [3]. However, Bekele et al. [6] found that teff protein consists of 3–15% prolamins. Adebowale et al. [7] found that prolamins, glutelins, and albumins + globulins accounted for 40%, 22%, and 11% of teff protein, respectively, and prolamin is its major storage protein. Shumoy et al. [8] reported that globulin contributes to 51% to 61% of total teff protein. Thus, the difference in protein fraction composition in teff, including both content and ratio, would be the main reason for its negative impact on the final product’s quality. 

To develop teff-based food products of acceptable quality, the functional properties of wheat gluten should be mimicked [9]. Thus, the difference in composition and structure between teff protein and wheat protein should be better understood. In this study, proteins were extracted from teff flour, and protein composition, structure, and properties were calculated and analyzed using wheat gliadin and glutenin as references, respectively, to broaden the utilization of teff and enrich the type of teff-based food products.

## 2. Materials and Methods

### 2.1. Materials

Whole-grain teff was bought from Bob’s Red Mill. It was milled with a high-speed grinder (YJ-200A, High-speed Multifunctional, Jinan, Shandong, China), sieved through an 80-mesh sieve, and used for this study. All chemicals used were of analytical grade (Sinopharm Chemical Reagent Factory, Co., Ltd., Shanghai, China).

### 2.2. Chemical Analysis

Moisture, fat, protein, starch, and ash in teff flour were determined using methods 925.10, 920.85, 979.09, 979.10, and 923.03 reported by AOAC, respectively. The crude protein content of teff flour was determined with the Kjeldahl method (protein conversion factors 5.4).

### 2.3. Protein Fraction and Quantification

After defatting with petroleum ether, teff flour was used for protein composition analysis using the modified Osborne method [10]. Briefly, teff flour (100 g) was stirred for 2 h with distilled water at a ratio of 1:5 (*w*/*v*) at 25 °C, and then the suspension was centrifuged to obtain teff albumin (6000× *g*, 4 °C, 10 min). The precipitate was re-extracted for 30 min with the same procedure described above. The supernatant was pooled, freeze-dried, and referred to as teff albumin. The residue gained after albumin extraction was resuspended and extracted twice with 1.25 M NaCl at 25 °C with the same procedure as the albumin. The suspension was centrifuged, pooled, and dialyzed against distilled water for 48 h at 4 °C. Then it was freeze-dried and referred to as teff globulin. After globulin was extracted, prolamin was extracted with the residue for 2 h with 500 mL of 60% (*v*/*v*) tert-butanol at 25 °C. The suspension was centrifuged (6000× *g*, 4 °C, 10 min). The extraction was repeated. The combined supernatant was concentrated with a rotating vacuum evaporator (RE-52C Model, Rotary Evaporator, Shanghai, China) at 40 °C, freeze-dried, and referred to as teff prolamin. The remaining residue was extracted twice for 1 h with 500 mL of 0.1 M NaOH at room temperature (22–25 °C) with the same procedure. Pooled supernatant was precipitated with TCA (final concentration: 10%). The suspension was centrifuged (6000× *g*, 4 °C, 10 min), and the pellet was dissolved in water, dialyzed, freeze-dried, and referred to as teff glutelin. Each protein fraction’s protein content (purity) was determined using the Kjeldahl method, and then the protein recovery rate was calculated.

### 2.4. Isoelectric Point Determination

The isoelectric point of each protein fraction was determined by isoelectric point precipitation [11]. In brief, fractioned proteins were resuspended in various extraction solutions, and their pH was adjusted to 7.0 (NaOH, 1 mol/L). Then hydrochloric acid (0.1%, *v*/*v*) was used for the pH adjustment of each protein solution. The concentration of protein in the supernatant was determined after centrifugation. The pH at which the supernatant showed the lowest protein concentration was taken as the isoelectric point of teff protein fractions. To minimize the denaturation effect caused by hydrochloric acid during isoelectric point precipitation, a modified method was developed based on the isolation of protein from rye and used for protein property determination [12]. With the isoelectric point determined, teff protein fractions were extracted, pH adjusted, and precipitated. They were dialyzed with a large amount of deionized water to remove acetic acid with stirring. Then, they were centrifuged with dialysis tubes with a molecular cut-off of 3500. The dialyzed suspension was lyophilized and used for further determination.

### 2.5. Molecular Size Determination

Sodium dodecyl sulfate–polyacrylamide gel electrophoresis (SDS-PAGE) was carried out on teff protein fractions according to the Laemmli method to determine their molecular size [13]. The discontinuous system composite with stacking gel and separator, with 5% and 12% acrylamide, respectively, was used. DTT (0.05% *w*/*v*) was used to reduce disulfide bonds in proteins in the reduction electrophoresis of the fractions. All samples were resuspended in sample buffer and heated at 100 °C for 10 min. Then, they were cooled and centrifuged (4500× *g*, 10 min), and the supernatants were loaded on the gels. A wide-range molecular weight marker between 15 and 130 kDa (Sigma Chemical Co., St. Louis, MO, USA) was used to determine the approximate molecular sizes of the proteins.

### 2.6. Amino Acid Analysis

The amino acid composition of the protein fractions was determined with an automatic amino acid analyzer (L-8900, Hitachi, Tokyo, Japan). Protein samples were hydrolyzed with 6 M HCl for 22 h at 120 °C in a sealed tube. Tryptophan was not determined in this study. The free energy of hydration (FEH) for each protein fraction was calculated according to amino acid composition [14].

### 2.7. Fourier Transfer Infrared (FTIR) Spectrometry

An FTIR (Cary 610/670, Varian Co., Palo Alto, CA, USA) was used for protein secondary structure analysis [15]. Protein samples (2 mg) were ground with KBr (200 mg), dried, and pressed into a sheet. Spectra were collected in the range of 4000–400 cm^−1^, and each spectrum was an average of 32 scans at 4 cm^−1^. OMINC 8.0 (Thermo Electron Co., Madison, WI, USA) and PeakFit 4.12 (SPSS Inc., Chicago, IL, USA) were used to deconvolve the spectra to quantitatively estimate the fraction of each band in Amide I.

### 2.8. Surface Hydrophobicity Determination

The surface hydrophobicity of protein fractions was determined using a fluorescent probe, 1-anilino-8-naphtalene-sulfonate (ANS), with minor modifications [16]. Protein fractions (0.500 g) were dispersed in a phosphoric acid buffer (0.01 mol/L, pH 7.0). They were magnetically stirred (1 h) and centrifuged (3500× *g*, 25 min). Determine protein concentration by the Coomassie Bright Blue G-250 method and then dilute the supernatant with PBS (pH 7.0) to obtain protein solutions with five different concentrations. A total of 4 mL protein solution was mixed with 20 μL ANS (8 mmol/L) solution, and a spectrofluorimetric assessment was carried out with an excitation wavelength of 390 nm and an emission wavelength of 470 nm. Curves were made by taking the protein concentration and the measured fluorescence intensity as the independent and corresponding variables, respectively. Then, curves were linearly fitted, and the fitted line was taken as the surface hydrophobicity index of the protein. 

### 2.9. Heat Stability of Protein Fractions

The heat stability of the protein fraction was determined using DSC (200-F3, NETZSCH, Selb, Bayern, Germany) and represented by its denaturing temperature (onset temperature and peak temperature). The protein samples (5.0 mg) and 10 µL phosphate buffer (0.05 M pH = 7.5) were added into aluminum pans and sealed and equilibrated for 3 h before the test. An empty aluminum pan was used as a reference. The determination was conducted between 25 and 120 °C at a heating rate of 10 °C/min and a nitrogen flow rate of 20.0 mL/min. The onset denaturation peak and peak denaturation temperatures were calculated and compared [8].

### 2.10. Statistical Analyses

The data are shown as mean ± SD. Origin 2022 (OriginLab Corporation, Northampton, MA, USA) was used for data treatment and diagram making. Statistical analyses were carried out using SPSS 21.0 (SPSS Inc., Chicago, IL, USA). All statistical analyses were performed by one-way analysis of variance with the Duncan post hoc test with a significance level of *p* < 0.05.

## 3. Results and Discussion

### 3.1. Composition of Teff Flour

The moisture, protein, starch, fat, and ash content of teff flour is shown in Table 1. They were consistent with previous studies [17,18]. It should be specified that data on teff protein content were reported in a wide range from 12.8 to 20.9% [15]. Here, the protein content of teff was determined to be 9.07%, which is similar to the 9.37% obtained by Shumoy et al. [8] and Gebru et al. [18]. The variation in the protein content would be caused by the varieties, cultivars, and cultivation conditions of the teff used. It has been demonstrated that white teff has a higher protein content than brown teff [17]. Methods used in protein extraction would also influence the results [18]. Moreover, various protein conversion factors were used by researchers [8]. In this study, the protein content was obtained with commercial flour. Thus, it can be used as guidance in industrial teff food development. 

### 3.2. Protein Content and Composition

After Osborne protein extraction, the protein content in each fraction (purity) was determined, and the relative content of each protein fraction was calculated subsequently. Results are shown in Table 2. It was observed that teff protein fractions showed low protein purity compared with that of wheat gliadin and glutenin extracted from wheat gluten. That is because the Osborne method is not a specific extraction method. It was carried out based on the solubility of ingredients in various types of solvents. Except for proteins, starch, pigments, and other similar solubility ingredients would also be extracted simultaneously with proteins. Results of protein relative content showed that teff prolamin was the main protein fraction of teff flour, followed by teff glutelin in this study. Teff albumin and teff globulin showed relatively low content. These results were similar to those of Adebowale et al. [7] but quite different from others reported previously [18,19]. Except for the cultivar and cultivation conditions discussed previously, the extraction method would also significantly affect the results. The lower teff prolamin content should be caused by the solvent used during teff prolamin extraction. A total of 50% propan-1-ol was suggested to replace 70% ethanol in prolamin extraction to minimize prolamin remaining in the residue. β-mercaptoethanol and a combination of 60% tert-butanol and 0.05% DTT were also used as more hydrophobic solvents in prolamin extraction, and their effectiveness was proved [7,18].

Prolamins constitute the main endosperm proteins in cereals, excluding oats and rice. These proteins provide most of the amino acids found in grains, significantly impacting their nutritional value for humans [19]. A balance between prolamin and glutelin is required for optimal cereal flour processing quality. The unique viscoelastic property of vital wheat gluten gives wheat flour incomparable processing properties in flour-based product development. Thus, the significant difference in protein composition between teff and wheat protein fractions would be one of the main reasons for the inferior processing properties of teff flour. Additionally, Zhang et al. [5] found that globulin could facilitate the weakening of the S–S bonds in the gluten network and cross-link with SDS-soluble gluten, promote the aggregation of protein molecules during cooking, and improve the end quality of cooked noodles. Thus, the high content of globulin in teff flour would also influence the processing properties of teff flour.

### 3.3. Isoelectric Point of Teff Protein Fractions

**Figure 1:**
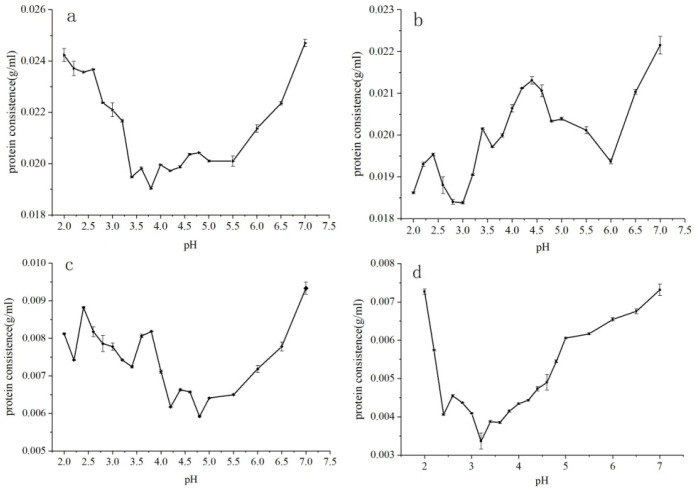
Isoelectric point of teff protein fractions. (**a**) Teff albumins; (**b**) teff globulins; (**c**) teff prolamins; and (**d**) teff glutelins.

Fractionate and reconstitution is a classical technique for analyzing the relationship between flour components and processing properties [4]. Flours were fractionated according to their component and water solubility, respectively, to obtain insight into the role of each fraction on the dough and final product properties [12]. In contrast, the purity of each fraction is the precondition for analyzing their underlying working mechanism during reconstitution. Thus, isoelectric point precipitation was conducted on teff protein fractions to improve their purity. Results in Figure 1 showed that the isoelectric points of teff albumin, globulin, prolamin, and glutelin were at pH 3.6, 3.0, 4.4, and 3.4, respectively. The isoelectric points of gliadin and glutenin in wheat gluten were reported at pH 5.5 and 4.6, respectively. Teff protein fractions had a lower PI when compared with wheat gluten fractions [20]. The pH of wheat dough was reported to be between 5.5 and 6.0 [21]. Thus, during dough formation, teff proteins performed better emulsifying activity because proteins had better solubility at a pH far away from their isoelectric point. But for teff globulin and glutelin, the higher solubility between pH 5.5 and 6.0 would lead to an inferior protein network, reflected by weak elasticity, decreased hardness during dough formation, and lower gas holding capacity during fermentation. 

### 3.4. Amino Acid Content of Teff Protein Fractions

The amino acid content in each protein fraction and their FEH were determined and calculated, and the results are shown in Table 3. Teff had a balanced essential and nonessential amino acid content, as reported previously [1]. Teff albumins and globulins were composited by a higher content of arginine, asparagine, and lysine, whereas they had a lower content of leucine, phenylalanine, glutamic acid/glutamine, and proline compared to teff prolamin and glutelin. Similar results were reported by Adebowale et al. [7]. To be specific, significantly lower leucine and proline were determined in teff prolamin compared to results published by Adebowale et al. [7], whereas their content in glutelins was almost the same. Compared with the results of Zhang et al. [22], teff globulin contained lower levels of all amino acids than wheat globulin, except tryptophan. It can be speculated that the extraction method of protein fractions influences not only the protein content but also the amino acid composition of protein fractions. Though teff prolamins and teff glutelins had significantly higher contents of proline than teff albumins and teff globulins, their contents were significantly lower than those of wheat gliadins and glutenins. Teff prolamins contained lower amounts of leucine and glutamic acid/glutamine than wheat gliadins, whereas teff prolamins and glutelins contained higher amounts of leucine and glutamic acid/glutamine than wheat glutenins. Based on information on teff flour’s amino acid and protein composition, it can be concluded that teff prolamin is its main storage protein.

The FEH for each protein fraction was calculated according to amino acid composition, and data on the FEH of each amino acid were published [19] (Table 3). Results of FEH showed that wheat glutenin FEH (−166.07 kcal/mol) was almost the same as that of wheat glutenin reported (165.81 kcal/mol), whereas wheat gliadin FEH (−146.84 kcal/mol) was much higher than the one reported (159.79 kcal/mol). In this study, the FEH for teff gliadin (−163.54 kcal/mol) was close to the value of teff prolamin reported, but the FEH for teff glutenin (−151.52 kcal/mol) was less negative than the one reported previously (−160.80 kcal/mol) [4,19]. These differences can also be attributed to the extraction method used. The FEH for teff prolamin (−163.54 kcal/mol) and globulin (−167.88 kcal/mol) was close to that of wheat glutenin but significantly more negative than teff albumin, teff glutelin, and wheat gliadin. Thus, teff prolamin was more hydrophobic than wheat gliadin.

### 3.5. Protein Molecular Weight Distribution of Teff Protein Fractions

**Figure 2:**
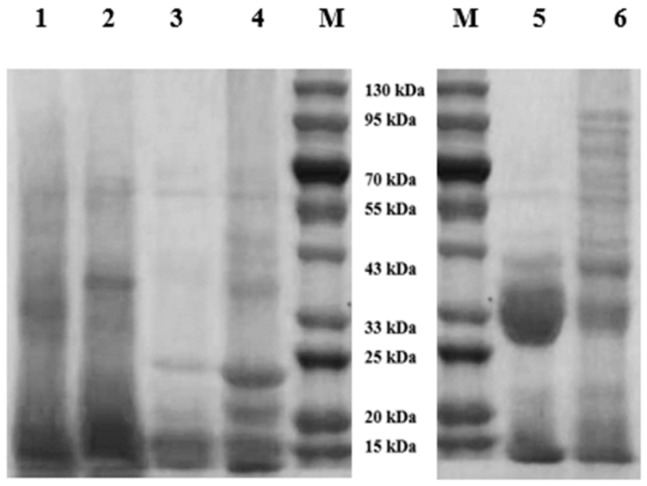
SDS-PAGE of protein fractions under reducing conditions. (**1**) Teff albumins; (**2**) teff globulins; (**3**) teff prolamins; (**4**) teff glutelins; (**5**) wheat gliadins; and (**6**) wheat glutenins, (**M**) maker.

The electrophoresis bands of protein fractions are shown in Figure 2. Though teff albumins showed poor definition, a broad molecular weight distribution can be observed compared to other teff protein fractions. It was similar to albumin fractioned by Gebru et al. [14]. The molecular weight of teff albumins was higher than that of wheat [23]. The molecular weight of albumins was related to the processing properties of dough [23]. Thus, differences in protein molecular weight distribution between teff albumins and wheat albumins could lead to differences in dough processing properties. Except for a very intense band between 33 kDa and 43 kDa, most teff globulins were observed below 25 kDa. The molecular weight of teff globulins was quite smaller than that of wheat globulins and buckwheat globulins [7,24]. Prolamins are characterized by their low molecular weights. Except for bands distinguished at 23 kDa and 19.5 kDa, proteins with molecular weights around 15 kDa were dominant in teff prolamins. It was in disagreement with the early report in which two major prolamin monomer bands at approximately 20.3 kDa and 22.8 kDa were found in teff, and they were considered to be the subunits of prolamin oligomers found in non-reducing SDS-PAGE [7]. The molecular weights of avenin and kafirin were reported to be distributed between 7 kDa and 34 kDa and 12 kDa and 27 kDa [25,26]. But for wheat gliadins, most proteins were distributed between 25 kDa and 43 kDa. The protein chain length of teff prolamins was smaller than that of gliadins, similar to avenin and kafirin. The molecular weight of dominant proteins in teff glutelins was below 25 kDa, whereas a high level of high molecular weight protein was shown in wheat glutenins. Wheat glutenins are divided into high molecular weight glutenin subunits (HMW-GS) and low molecular weight glutenin subunits (LMW-GS) according to the migration rate of the proteins during SDS-PAGE [27]. The proportion of HMW-GS was highly correlated with establishing the gluten network and dough strength. Thus, the lack of HMW-GS in teff and teff prolamin’s small chain length in teff glutelin posed a challenge for polymerization, resulting in ineffective protein networks and inferior processing properties of teff flour.

### 3.6. Secondary Structure of Teff Proteins

FTIR was used to analyze the secondary structure of proteins. The varied regions of amide I were assigned to β-sheets (1600–1640 cm^−1^), random coils (1640–1650 cm^−1^), α-helices (1650–1660 cm^−1^), and β-turns (1660–1700 cm^−1^), respectively. In all protein fractions, β-sheet was the predominant secondary structure, followed by β-turn (Table 4). Teff prolamin and wheat gliadin had lower β-sheet content, implying their lower structural stability [28]. It is reported that plant seed globulins are a class of β-sheet proteins [29], consistent with our findings on the secondary structures of teff globulins. The contents of random coils and α-helices in protein fractions were much different. Teff albumin and globulin showed similar secondary structures, implying similar interior conformations. Different from wheat gliadin, teff prolamin has the highest α-helices content (27.08%), whereas no random coils were determined. No significant differences were found in the content of the β-sheet between teff glutelin and wheat glutenin. No α-helice was determined in teff glutelin, and no random coil was determined in wheat glutenin. α-Helices take part in the formation of higher-order structures by burying the hydrophobic surface [30]. Thus, the lower α-helices and higher random coils in teff glutelin implied loosened protein structures, and the high α-helices in teff prolamin induced higher-order protein structures. 

### 3.7. Sulfhydryl and Disulfide Bond Contents and Surface Hydrophobicity of Teff Protein Fractions

**Figure 3:**
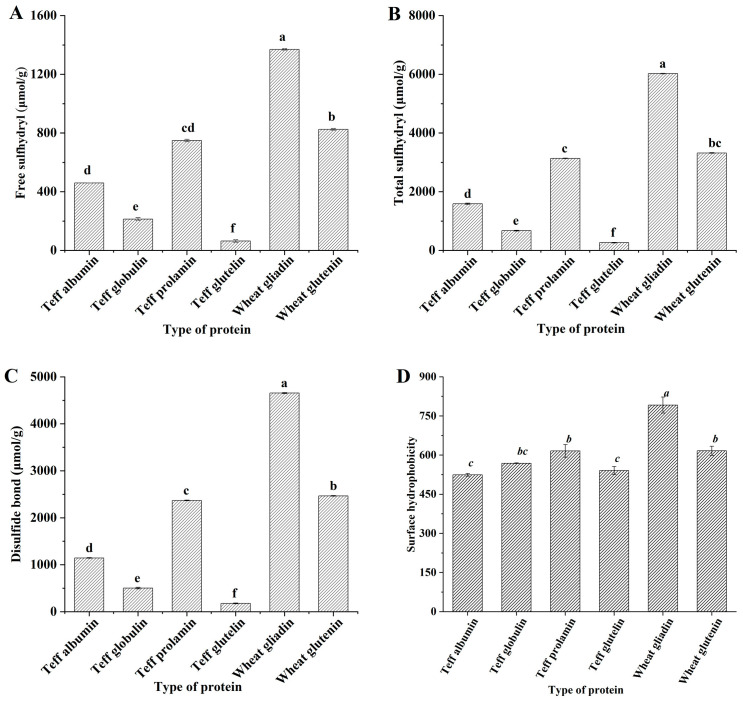
Free sulfhydryl (**A**), total sulfhydryl (**B**), disulfide bond (**C**), and surface hydrophobicity (**D**) of teff protein fractions. The values with different lower-case letters are significantly different (*p* < 0.05).

The content of sulfhydryl and disulfide bonds in each protein fraction is shown in Figure 3. Teff prolamins had the highest sulfhydryl and disulfide bond content in teff protein fractions, with teff glutelin having the lowest level. When compared with wheat gluten fractions, significant differences can be found, especially for the total sulfhydryl and disulfide bonds. It was generally accepted that disulfide bonds are crucial for wheat gluten proteins’ structure, properties, and functionality [31]. Teff prolamin, wheat gliadin, and wheat glutenin had high disulfide bond content and concomitantly low free sulfhydryl content in this study. To be specific, teff glutelin’s sulfhydryl and disulfide bond content was the lowest among all protein fractions. It was reported that the wheat gliadin proteins contained mostly intramolecular disulfide bonds [31]. The HWM-GS was formed by the disulfide linkage of several diverse polypeptide chains, and these linkages resulted in the unique viscoelasticity of wheat glutenin. Breaking the disulfide bond into sulfhydryl in noodles led to a rougher and looser cross-section and surface, as well as poor cooking and textural properties [32]. Thus, the lower content of the disulfide bond of teff prolamin and glutelin would be the reason for the poor functionality of teff flour. The surface hydrophobicity of proteins showed a more significant influence on functionality than the total hydrophobicity of proteins because proteins are macromolecular and work in a three-dimensional structure [33]. The protein surface hydrophobicity depended mainly on the hydrophobic residues exposed to the surface of the protein molecules. It was influenced by the molecular size and shape of proteins, amino acid content, and the secondary structure of proteins [34]. It affected the interaction between proteins and other macromolecules and between proteins and small ligands, influenced the formation of protein networks, and was closely related to protein surface tension, interfacial tension, and emulsifying activity [35]. Thus, it was usually used to indicate gluten properties during food processing [33,36]. The surface hydrophobicity of each protein fraction is shown in Figure 2. Teff albumin and glutelin had significantly lower surface hydrophobicity than teff prolamin. No significant differences were observed between the surface hydrophobicity of teff globulin and prolamin. The surface hydrophobicity of wheat gliadin and glutenin was significantly higher when compared with that of teff prolamin and glutelin, respectively. During dough formation, higher surface hydrophobicity implied more intramolecular or intermolecular cross-links between proteins and higher stability of protein networks. Wang et al. [36] have related the decrease in wheat gliadin surface hydrophobicity during frozen storage to the reduction in the amphipathic and foaming properties of gliadin. Thus, the lower surface hydrophobicity of teff prolamin and glutelin can be related to the poor dough-forming properties of teff flour.

### 3.8. Heat Stability of Teff Composite Proteins

The heat stability of protein fractions was determined with DSC and represented by onset temperature and peak temperature, respectively. Endothermic peaks at 58 °C for wheat gliadins and two endothermic peaks at 64 and 84 °C for wheat glutenins were reported at low water content [37]. When glutenin and gliadins were treated above 55 °C and 70 °C, respectively, proteins aggregated via disulfide bonds [18]. Thus, protein structure changes irreversibly during thermal processing, known as “protein thermal denaturation.” In this study, significantly lower endothermic peaks were observed for wheat gliadin when compared with teff prolamin. Table 5 shows that teff prolamin had the highest endothermic temperature, accompanied by the biggest denaturation temperature range compared with other protein fractions. The onset and peak temperatures of teff glutelin were significantly lower than those of wheat glutenin. The heat stability of protein fractions reflects the structural properties of proteins and influences the end quality of flour products. After thermal denaturation, the viscoelasticity of gluten changed. The ratio of protein to thermal denaturation is indicative of the protein’s resistance to thermal stability [22]. Kovacs et al. [38] found that the low ratio of monomeric proteins, such as gliadins, as well as the high ratio of LWM-GS to HMW-GS in wheat glutenin resulted in low thermal stability of the gluten. In this study, the high prolamin content, high onset and peak temperatures, and low ratio of LWM-GS to HMW-GS in teff would result in high thermal stability and slow denaturation of proteins in teff dough during cooking. Slow denaturation allowed the disintegration of the noodle structure during cooking by accelerating hydration and promoting extensive disintegration of starch granules [38].

## 4. Conclusions

To develop teff-based food products of acceptable quality, the functional properties of gluten should be mimicked. This study demonstrated that teff flour contained 9.07% protein, with teff prolamin as its primary protein fraction. The isoelectric points of albumin, globulin, prolamin, and glutelin were at pH 3.6, 3.0, 4.4, and 3.4, respectively. Teff prolamins and glutelins showed a significant difference in proline, leucine, and glutamic acid/glutamine content compared to wheat gliadins and glutenins. The FEH for teff prolamin and globulin was close to that of wheat glutenin but significantly more negative than teff albumin, teff glutelin, and wheat gliadin. The protein chain length of teff prolamins was smaller than that of wheat gliadins, and teff glutelins had a lack of high molecular weight glutenin. Teff prolamin had higher-order protein structures, while teff glutenin had loosened protein structures. Teff prolamin and glutelin had lower disulfide bond contents and surface hydrophobicity. Teff prolamin had significantly higher thermal stability than wheat gliadin, whereas the thermal stability of teff glutelin was significantly lower than that of wheat glutenin. The poor processing properties of teff flour may be interpreted, and strategies could be designed with protein recombination in future work. Glutenin, especially its HMW-GS, is promising to be used to improve the protein networks formed in teff dough during dough preparation and improve teff-based food quality. Except for the teff flour used in this study, other commercial teff flour would be provided globally. Further studies would be conducted to compare their processing quality and choose the optimal one according to the product type.

## Figures and Tables

**Table 1:** Composition of teff flour.

Fraction	Moisture, g/100 g	Protein, g/100 g	Starch, g/100 g	Fat, g/100 g	Ash, g/100 g
Content	10.68 ± 0.42	9.07 ± 0.03	72.57 ± 0.09	2.20 ± 0.17	2.54 ± 0.10

**Table 2:** Purity and relative protein content of protein fractions.

Fraction	Purity, %	Protein Relative Content, % *	Recovery %
Teff albumin	18.28 ± 0.06	18.05 ± 0.06	83.87 ± 0.01
Teff globulin	63.76 ± 0.05	19.73 ± 0.05	
Teff prolamin	42.34 ± 0.07	33.47 ± 0.02	
Teff glutelin	37.14 ± 0.03	28.75 ± 0.07	
Wheat gliadin	73.71 ± 0.03	37.65 ± 0.09	97.70 ± 0.16
Wheat glutenin	52.92 ± 0.03	62.35 ± 0.07	

* The relative protein content of teff protein fractions was based on teff total protein, and the relative content of wheat gliadin and wheat glutenin was based on wheat gluten.

**Table 3:** The amino acid content of teff and gluten protein fractions.

Amino Acids	Amino Acids Content (g/100 g)						Free Energy of Hydration (kcal/mol)						
	Teff Albumin	Teff Globulin	Teff Prolamin	Teff Glutelin	Wheat Gliadin	Wheat Glutenin	Amino Acid	Teff Albumin	Teff Globulin	Teff Prolamin	Teff Glutelin	Wheat Gliadin	Wheat Glutenin
Lys	3.94	2.42	0.26	1.47	1.04	3.12	−3.77	−27.44	−20.39	−1.75	−6.96	−3.67	−12.64
Thr	2.41	1.64	2.23	2.75	2.31	2.56	−1.69	−9.23	−7.60	−8.26	−7.16	−4.48	−5.71
Ile	2.57	1.62	2.29	3.20	4.75	2.09	0.07	0.37	0.28	0.32	0.31	0.35	0.18
Leu	4.65	3.60	5.09	6.84	9.32	5.19	0.07	0.67	0.63	0.71	0.67	0.68	0.44
Tyr	2.85	2.50	3.38	4.56	3.12	2.40	−2.82	−11.98	−12.71	−13.74	−13.03	−6.64	−5.87
Phe	2.36	1.89	3.05	4.20	4.60	2.95	−0.28	−1.08	−1.05	−1.35	−1.31	−1.07	−0.79
Val	3.04	2.49	2.90	3.80	10	4.11	0.04	0.28	0.28	0.26	0.24	0.24	0.22
Met	0.86	0.86	0.00	2.20	1.61	1.17	−0.10	−0.16	−0.19	0.00	−0.27	−0.15	−0.12
His	2.40	1.26	0.77	1.53	2.89	3.29	−2.18	−9.11	−5.78	−2.83	−3.95	−5.55	−7.26
Ala	3.71	2.86	2.74	4.63	2.55	3.75	−0.06	−0.67	−0.63	−0.48	−0.57	−0.23	−0.40
Arg	3.78	3.50	0.51	1.91	3.99	7.64	−6.85	−40.14	−44.96	−5.24	−13.79	−21.44	−47.20
Asp	4.24	2.55	1.60	3.04	3.44	5.92	−3.11	−26.76	−19.47	−9.76	−13.04	−10.99	−21.73
Glu	2.04	5.83	19.77	20.58	26.6	15.93	−3.15	−11.80	−40.78	−110.53	−80.90	−77.83	−53.58
Gly	2.94	2.29	0.82	1.95	2.13	4.61	−0.23	−2.43	−2.29	−0.66	−1.10	−0.89	−2.22
Ser	2.28	1.96	2.04	3.06	6.31	3.76	−2.36	−13.83	−14.38	−11.96	−12.62	−19.37	−13.26
Pro	2.41	1.78	3.33	5.36	15.35	12.33	0.23	1.30	1.16	1.74	1.97	4.19	3.87
Total amino acid	44.07	37.27	47.45	65.72	79.76	68.49	Total	−152.00	−167.88	−163.54	−151.52	−146.84	−166.07
Essential amino acid %	25.08 (132)	18.28 (96)	19.97 (73)	30.55 (87)	34.74 (77)	26.88 (65)							
Nonessential amino acid %	18.99 (57)	18.99 (49)	27.48 (42)	35.17 (46)	45.02 (44)	41.61 (39)							

**Table 4:** Secondary structure of teff protein fractions.

	Teff Albumin	Teff Globulin	Teff Prolamin	Teff Glutelin	Wheat Gliadin	Wheat Glutenin
α-helice (%)	10.35 ± 0.01 ^d^	10.40 ± 0.01 ^d^	27.08 ± 0.01 ^a^	-	19.9 ± 0.01 ^b^	13.02 ± 0.01 ^c^
β-sheet (%)	54.28 ± 0.05 ^a^	53.89 ± 0.03 ^b^	43.45 ± 0.02 ^e^	53.50 ± 0.02 ^c^	46.82 ± 0.04 ^d^	53.35 ± 0.01 ^c^
β-turn (%)	23.86 ± 0.01 ^e^	24.81 ± 0.01 ^d^	29.48 ± 0.02 ^c^	32.34 ± 0.00 ^b^	22.41 ± 0.01 ^f^	33.63 ± 0.01 ^a^
Random coil (%)	11.51 ± 0.05 ^b^	11.52 ± 0.01 ^b^	-	14.15 ± 0.01 ^a^	10.87 ± 0.01 ^c^	-

Different letters in the same row indicate significant differences (*p* < 0.05).

**Table 5:** Heat stability of teff protein fractions.

	Teff Albumin	Teff Globulin	Teff Prolamin	Teff Glutelin	Wheat Gliadin	Wheat Glutenin
Onset temperature (°C)	42.17 ± 0.15	50.50 ± 0.020	65.20 ± 0.20	41.90 ± 0.15	41.70 ± 0.20	52.30 ± 0.15
Peak temperature (°C)	46.37 ± 0.15	57.09 ± 0.11	78.33 ± 0.15	46.50 ± 0.05	45.00 ± 0.20	60.20 ± 0.02

## Data Availability

All data are available from the corresponding author upon reasonable request.

## References

[1] Nascimento K.d.O.d., Paes S.d.N.D., Oliveira I.R.d., Reis I.P., Augusta I.M. (2018). Teff: Suitability for Different Food Applications and as a Raw Material of Gluten-free, a Literature Review. J. Food Nutr. Res..

[2] Alaunyte I., Stojceska V., Plunkett A., Ainsworth P., Derbyshire E. (2012). Improving the quality of nutrient-rich Teff (Eragrostis tef) breads by combination of enzymes in straight dough and sourdough breadmaking. J. Cereal Sci..

[3] Wieser H., Koehler P., Scherf K.A. (2023). Chemistry of wheat gluten proteins: Qualitative composition. Cereal Chem..

[4] Wrigley C., Békés F., Bushuk W. (2006). Gluten: A balance of gliadin and glutenin. Gliadin and Glutenin: The Unique Balance of Wheat Quality.

[5] Zhang L.L., Guan E.Q., Yang Y.L., Liu Y.X., Zhang T.J., Bian K. (2021). Impact of wheat globulin addition on dough rheological properties and quality of cooked noodles. Food Chem..

[6] Bekele E. (1995). Essential and non-essential amino acids in a free state and in the major protein fractions of teff seeds. SINET Ethiop. J. Sci..

[7] Adebowale A.-R.A., Emmambux M.N., Beukes M., Taylor J.R. (2011). Fractionation and characterization of teff proteins. J. Cereal Sci..

[8] Shumoy H., Pattyn S., Raes K. (2018). Tef protein: Solubility characterization, in-vitro digestibility and its suitability as a gluten free ingredient. LWT Food Sci. Technol..

[9] Taylor J.R.N., Taylor J., Campanella O.H., Hamaker B.R. (2016). Functionality of the storage proteins in gluten-free cereals and pseudocereals in dough systems. J. Cereal Sci..

[10] Osborne T.B., Voorhees C.L. (1894). Proteids of the wheat kernel. J. Am. Chem. Soc..

[11] Novák P., Havlíček V., Ciborowski P., Silberring J. (2016). 4—Protein extraction and precipitation. Proteomic Profiling and Analytical Chemistry.

[12] Grossmann I., Koehler P. (2016). Fractionation-reconstitution studies to determine the functional properties of rye flour constituents. J. Cereal Sci..

[13] Laemmli U. (1970). Cleavage of Structural Proteins during the Assembly of the Head of Bacteriophage T4. Nature.

[14] Shewry P., Halford N., Tatham A., Popineau Y., Lafiandra D., Belton P. (2003). The high molecular weight subunits of wheat glutenin and their role in determining wheat processing properties. Adv. Food Nutr. Res..

[15] Byler D.M., Susi H. (1986). Examination of the secondary structure of proteins by deconvolved FTIR spectra. Biopolym. Orig. Res. Biomol..

[16] Cardamone M., Puri N.K. (1992). Spectrofluorimetric assessment of the surface hydrophobicity of proteins. Biochem. J..

[17] Bultosa G. (2007). Physicochemical characteristics of grain and flour in 13 Tef (Eragrostis tef (Zucc.) Trotter) grain varieties. J. Appl. Sci. Res..

[18] Gebru Y.A., Hyun-Ii J., Young-Soo K., Myung-Kon K., Kwang-Pyo K. (2019). Variations in Amino Acid and Protein Profiles in White versus Brown Teff (Eragrostis Tef) Seeds, and Effect of Extraction Methods on Protein Yields. Foods.

[19] Labuschagne M.T. (2018). A review of cereal grain proteomics and its potential for sorghum improvement. J. Cereal Sci..

[20] Liu J., Luo D., Li X., Xu B., Zhang X., Liu J. (2016). Effects of inulin on the structure and emulsifying properties of protein components in dough. Food Chem..

[21] Verheyen C., Jekle M., Becker T. (2014). Effects of Saccharomyces cerevisiae on the structural kinetics of wheat dough during fermentation. LWT Food Sci. Technol..

[22] Zhang L.L., Li M.M., Guan E.Q., Yang Y.L., Zhang T.J., Liu Y.X., Bian K. (2022). Interactions between wheat globulin and gluten under alkali or salt condition and its effects on noodle dough rheology and end quality. Food Chemistry.

[23] Tomić J., Torbica A., Popović L., Strelec I., Vaštag Ž., Pojić M., Rakita S. (2015). Albumins characterization in relation to rheological properties and enzymatic activity of wheat flour dough. J. Agric. Sci. Technol..

[24] Guo X.N., Yao H.Y. (2006). Fractionation and characterization of tartary buckwheat flour proteins. Food Chem..

[25] Kumar L., Sehrawat R., Kong Y. (2021). Oat proteins: A perspective on functional properties. LWT.

[26] Belton P.S., Delgadillo I., Halford N.G., Shewry P.R. (2006). Kafirin structure and functionality. J. Cereal Sci..

[27] Shewry P.R., Lafiandra D. (2022). Wheat glutenin polymers 1. structure, assembly and properties. J. Cereal Sci..

[28] Zeng H.-Y., Cai L.-H., Cai X.-L., Wang Y.-J., Li Y.-Q. (2011). Structure characterization of protein fractions from lotus (Nelumbo nucifera) seed. J. Mol. Struct..

[29] Zirwer D., Gast K., Welfle H., Schlesier B., Dieter Schwenke K. (1985). Secondary structure of globulins from plant seeds: A re-evaluation from circular dichroism measurements. Int. J. Biol. Macromol..

[30] Efimov A.V. (1999). Complementary packing of α-helices in proteins. FEBS Lett..

[31] Huebner F.R., Bietz J.A., Wall J.S., Friedman M. (1977). Disulfide bonds: Key to wheat protein functionality. Protein Crosslinking: Biochemical and Molecular Aspects.

[32] Li C.C., Lu Q.Y., Liu Z.P., Yan H.L. (2018). Effects of the Addition of Gluten with Different Disulfide Bonds and Sulfhydryl Concentrations on Chinese White Noodle Quality. Czech J. Food Sci..

[33] Chandrapala J., Zisu B., Palmer M., Kentish S., Ashokkumar M. (2011). Effects of ultrasound on the thermal and structural characteristics of proteins in reconstituted whey protein concentrate. Ultrason. Sonochemistry.

[34] Jiang L., Wang Z., Li Y., Meng X., Sui X., Qi B., Zhou L. (2015). Relationship Between Surface Hydrophobicity and Structure of Soy Protein Isolate Subjected to Different Ionic Strength. Int. J. Food Prop..

[35] Nakai S., Ho L., Helbig N., Kato A., Tung M.A. (1980). Relationship Between Hydrophobicity and Emulsifying Properties of Some Plant Proteins. Can. Inst. Food Sci. Technol. J..

[36] Wang P., Tao H., Wu F., Yang N., Chen F., Jin Z., Xu X. (2014). Effect of frozen storage on the foaming properties of wheat gliadin. Food Chem..

[37] León A., Rosell C.M., de Barber C.B. (2003). A differential scanning calorimetry study of wheat proteins. Eur. Food Res. Technol..

[38] Kovacs M.I.P., Fu B.X., Woods S.M., Khan K. (2004). Thermal stability of wheat gluten protein: Its effect on dough properties and noodle texture. J. Cereal Sci..

